# Parasite effectors target helper NLRs in plants to suppress immunity-related cell death

**DOI:** 10.1371/journal.pbio.3001395

**Published:** 2021-09-17

**Authors:** Yuanyuan Li, Nathan Meier, Savithramma P. Dinesh-Kumar

**Affiliations:** Department of Plant Biology and The Genome Center, College of Biological Sciences, University of California, Davis, California, United States of America

## Abstract

For successful colonization, parasites need to target the plant immune system. This Primer explores a new study in PLOS Biology which reveal that unrelated parasites have evolved effectors which specifically suppress the function of helper NLRs, explaining the complex plant-parasite coevolutionary dynamics.

## Teaser

Parasites target the plant immune system for successful colonization. A new study in *PLOS Biology* reveals that unrelated parasites have evolved effectors that specifically suppress the function of helper nucleotide-binding leucine-rich repeats (NLRs), explaining the complex plant–parasite coevolutionary dynamics.

**Fig 1:**
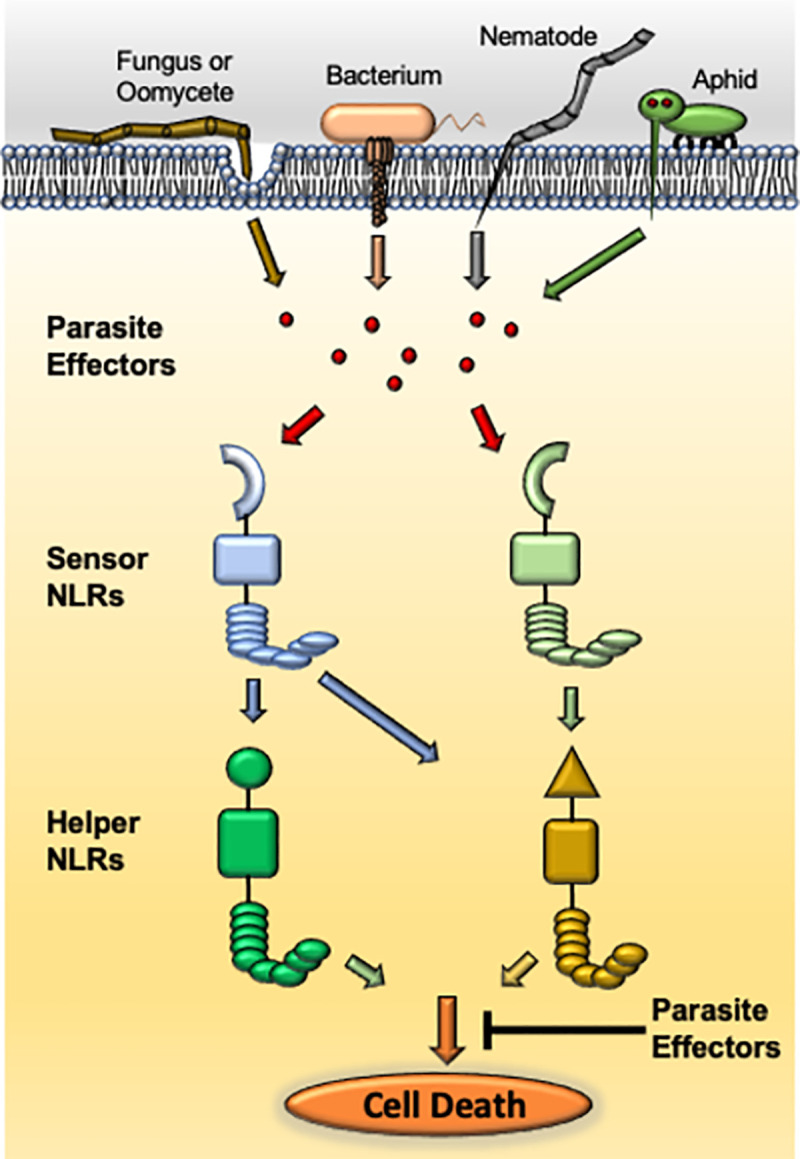
Suppression of NLR immune receptor–mediated cell death by parasitic effectors. Plants recognize effectors secreted from evolutionarily unrelated parasites such as bacteria, fungi, oomycetes, nematodes, aphids, and others via sensor NLRs. This recognition event activates downstream immune signaling leading to cell death, which requires the function of one or more helper NLRs. Derevnina and colleagues [8] used an effectoromics approach to show that parasite effectors interfere with helper NLRs function and suppress the immunity-related cell death response. NLR, nucleotide-binding leucine-rich repeat.

Plants have evolved a robust innate immune system to defend against a large number of parasites present in their ecological niche [1]. The plant’s first layer of defense involves detection of conserved pathogen/microbe-associated molecular patterns (PAMPs/MAMPs) leading to pattern-triggered immunity (PTI). Parasites have evolved to sense and suppress PTI by secreting virulence factors called effectors into plant cells. Plants have evolved to recognize parasite-encoded effectors via the nucleotide-binding leucine-rich repeat (NLR) class of immune receptors and activate more robust effector-triggered immunity (ETI), which ultimately leads to cell death at the site of infection called the hypersensitive response (HR) [1] (Fig 1). This serves to starve out parasites and prevent further colonization.

The plant genome encodes a large number of NLRs [2,3]. The NLRs that are involved in the recognition of parasite effectors either directly or indirectly are classified as sensor NLRs [2,4,5] (Fig 1). Accumulating evidence indicates that some sensor NLRs require the function of other NLRs, referred to as helper NLRs, to activate downstream immune signaling and cell death induction [4,5] (Fig 1). To date, there are 3 described helper NLR families: the Activated Disease Resistance 1 (ADR1) family, the N Requirement Gene 1 (NRG1) family, and NB-LRR protein required for HR-associated cell death (NRC) family [6].

The Solanaceae-specific NRC family is required for the function of a large number of CC-type sensor NLRs that confer resistance to a diverse array of parasites [4,5]. An emerging model shows that NRCs and their sensor NLR partners form a complex genetic network, in which NRCs form redundant central nodes and also exhibit some specificity toward their sensor NLR partners. For example, the sensor NLR Prf requires NRC2 and NRC3 to confer resistance to *Pseudomonas syringae* bacteria, but NLR Rpi-blb2 only requires NRC4 to confer resistance to potato blight caused by the oomycete pathogen *Phytophthora infestans* [4,5]. Furthermore, Rx requires NRC2, NRC3, and NRC4 in a genetically redundant manner to confer resistance to *Potato virus X* (PVX). Given that some helper NLRs are points of convergence in downstream signaling of multiple upstream sensor NLRs, they would be ideal targets for parasite effectors to overcome NLR-mediated ETI.

In this issue of *PLOS Biology*, the Kamoun group [7] provide some of the first evidence of parasitic effectors interfering with helper NLR–mediated cell death (Fig 1). Using an effectoromics screen with a library consisting of a total of 165 effectors from bacteria, oomycete, nematode, and aphid, they have identified 5 effectors that can suppress the cell death induced by 2 NRC-dependent sensor NLRs, Prf or Rpi-blb2, in *Nicotiana benthamiana* plants. Among these effectors, SS15, from the cyst nematode *Globodera rostochiensis*, and AVRcap1b, from the potato late blight oomycete pathogen *P*. *infestans*, showed strong suppression of the cell death triggered by autoimmune mutants of NRC2 and NRC3, but not NRC4. This demonstrated that these 2 effectors act specifically at the level of the NRC2 and NRC3 helpers or their downstream pathways, independent of their upstream sensor NLR partners. In agreement with these findings, these 2 effectors can suppress sensor NLR, Rx-mediated cell death in response to PVX only if NRC4 is silenced.

Using a combination of yeast two-hybrid (Y2H), *in vitro* and *in planta* assays, the Kamoun group [7] revealed that SS15 and AVRcap1b effectors may suppress NRC2 and NRC3 functions through distinct mechanisms. The SS15 effector directly associates with NRC2 and NRC3 through the NB-ARC domain. Furthermore, SS15 can bind both inactive and autoactive forms of NRC2 and NRC3. On the other hand, AVRcap1b does not associate directly with NRCs; instead, it interacts with another host protein, NbTOL9a (Target of Myb 1-like protein 9a). Silencing of NbTOL9a enhances cell death induced by autoactive NRC2 and NRC3, but not NRC4, indicating that NbTOL9a functions as a negative regulator of cell death. These findings suggested that AVRcap1b effector co-opts NbTOL9a from the host to suppress NRC2- and NRC4-mediated cell death.

The findings by Derevnina and colleagues [8] provide further insights into the evolutionary arms race between parasites and hosts. Although plants have evolved with complex NLR immune receptor networks to defend against parasites, parasites have evolved effectors that counteract central nodes of these networks. The study also reinforces the importance of using parasite effectors as probes to dissect complex immune signaling networks in plants. Effectoromics approaches involving other effectors from diverse parasites should uncover additional effectors that interfere with NRCs including NRC4 and other helper NLRs.

Additional studies are needed to understand the mechanisms by which SS15 and AVRcap1b interfere with helper NLRs function. How does SS15 binding to the NB-ARC domain of NRC2 and NRC3 suppress cell death? Since there is no evidence so far for direct interaction between NRCs and corresponding sensor NLR partners, does SS15 binding interfere with downstream signaling steps that are required for cell death execution? Recent studies uncovered that the activated sensor NLR, ZAR1, and the helper-NLRs, NRG1, and ADR1 function as cation channels that promote calcium influx leading to HR cell death [8,9]. Since the N-terminal MADA motif of NRCs, which is sufficient to induce cell death, is functionally conserved in ZAR1 and the larger MADA-CC-NLR family [10], NRCs are more likely to function through similar mechanisms by forming cation channels. It will be interesting to test if the binding of SS15 to the NB-ARC domain interferes with channel formation and/or function, leading to the suppression of cell death. With respect to AVRcap1b effector, considering that both AVRcap1b and its interacting partner, NbTOL9a, do not associate directly with NRCs, how does the AVRcap1b-NbTOL9a complex interfere with NRC-mediated cell death? Answers to these questions should improve our understanding of the role NRCs and other helper NLRs play in ETI.

## Abbreviations

ADR1: Activated Disease Resistance 1
ETI: effector-triggered immunity
HR: hypersensitive response
NLR: nucleotide-binding leucine-rich repeat
NRC: NB-LRR protein required for HR-associated cell death
NRG1: N Requirement Gene 1
PAMP/MAMP: pathogen/microbe-associated molecular pattern
PTI: pattern-triggered immunity
PVX: *Potato virus X*
Y2H: yeast two-hybrid

## References

[1] Zhou JM, Zhang Y. Plant Immunity: Danger Perception and Signaling. Cell. 2020;181(5):978–89. doi: 10.1016/j.cell.2020.04.028 . 32442407

[2] Baggs E, Dagdas G, Krasileva KV. NLR diversity, helpers and integrated domains: making sense of the NLR IDentity. Curr Opin Plant Biol. 2017;38:59–67. doi: 10.1016/j.pbi.2017.04.012 . 28494248

[3] Adachi H, Derevnina L, Kamoun S. NLR singletons, pairs, and networks: evolution, assembly, and regulation of the intracellular immunoreceptor circuitry of plants. Curr Opin Plant Biol. 2019;50:121–31. doi: 10.1016/j.pbi.2019.04.007 . 31154077

[4] Wu CH, Abd-El-Haliem A, Bozkurt TO, Belhaj K, Terauchi R, Vossen JH, et al. NLR network mediates immunity to diverse plant pathogens. Proc Natl Acad Sci U S A. 2017;114(30):8113–8. doi: 10.1073/pnas.1702041114 . 28698366 PMC5544293

[5] Wu CH, Derevnina L, Kamoun S. Receptor networks underpin plant immunity. Science. 2018;360(6395):1300–1. doi: 10.1126/science.aat2623 . 29930125

[6] Jubic LM, Saile S, Furzer OJ, El Kasmi F, Dangl JL. Help wanted: helper NLRs and plant immune responses. Curr Opin Plant Biol. 2019;50:82–94. doi: 10.1016/j.pbi.2019.03.013 . 31063902

[7] Derevnina L, Contreras MP, Adachi H, Upson J, Cruces AV, Xie R, et al. Plant pathogens convergently evolved to counteract redundant nodes of an NLR immune receptor network. PLoS Biol. 2021. doi: 10.1371/journal.pbio.3001136 34424903 PMC8412950

[8] Bi G, Su M, Li N, Liang Y, Dang S, Xu J, et al The ZAR1 resistosome is a calcium-permeable channel triggering plant immune signaling. Cell. 2021;184(13):3528–41.e12. doi: 10.1016/j.cell.2021.05.003 33984278

[9] Jacob P, Kim NH, Wu F, El-Kasmi F, Chi Y, Walton WG, et al. Plant "helper" immune receptors are Ca(2+)-permeable nonselective cation channels. Science. 2021;373:420–425. doi: 10.1126/science.abg7917 . 34140391 PMC8939002

[10] Adachi H, Contreras MP, Harant A, Wu CH, Derevnina L, Sakai T, et al. An N-terminal motif in NLR immune receptors is functionally conserved across distantly related plant species. Elife. 2019;8. doi: 10.7554/eLife.49956. 31774397 PMC6944444

